# Intravascular Ultrasound in the Endovascular Treatment of Patients With Peripheral Arterial Disease: Current Role and Future Perspectives

**DOI:** 10.3389/fcvm.2020.551861

**Published:** 2020-12-02

**Authors:** Romaric Loffroy, Nicolas Falvo, Christophe Galland, Léo Fréchier, Frédérik Ledan, Marco Midulla, Olivier Chevallier

**Affiliations:** Department of Vascular Medicine and Interventional Radiology, François-Mitterrand University Hospital, Dijon, France

**Keywords:** intravascular ultrasound (IVUS), peripheral arterial disease, percutaneous transluminal angioplasty (PTA), stent placement, atherectomy

## Abstract

Over the last decade, intravascular ultrasound (IVUS) has emerged as a useful adjunctive tool to angiography in an increasing number of catheter-based procedures for peripheral arterial disease (PAD). IVUS catheters offer accurate cross-sectional imaging of arterial vessels with high dimensional accuracy and provide accurate information about lesion morphology. IVUS enables assessment of the plaque morphology, vessel diameter, and the presence of arterial dissections. Furthermore, IVUS is able to properly guide the best choice of appropriate percutaneous transluminal angioplasty (PTA) technique, guide the delivery of different devices, and assess the immediate result of any endovascular intervention. In the present review, the role of IVUS for PAD will be discussed, specifically the applications of IVUS technology during interventional procedures including PTA, stent sizing, crossing total occlusion, assessing residual narrowing and stent apposition and expansion, and atherectomy. Future perspectives of IVUS-guided treatments and cost-effectiveness of the systematic use of IVUS during endovascular interventions will be also discussed.

## Introduction

Advances in endovascular techniques and devices have led to the increased use of percutaneous procedures to treat peripheral arterial disease (PAD) over the last decade (1). Smaller delivery devices, better quality materials for balloons and stents, more predictable delivery mechanisms, increased familiarity among vascular specialists using microguide wires, and the ever-aging population at risk have led to an important increase in the number of vascular interventions being performed (2). Because of this increase in the volume of procedures performed, more emphasis should be placed on precise imaging modalities to guide such procedures. Digital subtraction angiography (DSA) remains a widely used imaging modality during endovascular peripheral procedures and is still the gold standard (2). However, traditional DSA has its limitations by underestimating several morphological aspects of atherosclerotic lesions and by its ability to only display the outline of the vessel lumen.

Intravascular ultrasound (IVUS) has emerged as an important adjunctive modality to DSA in the guidance of coronary percutaneous transluminal angioplasty (PTA) (3). However, its use for peripheral applications is less popular (4–6). IVUS is the ideal imaging tool to help guide peripheral endovascular procedures because of its ease and accuracy at determining different imaging parameters, including luminal cross-sectional measurements and accurate information about lesion morphology such as true vessel diameters, wall thickness/layers, length, shape, and volume of lesion, position of lesion within the lumen (concentric or eccentric), type of lesion (fibrous, necrotic, calcified and mixed), any presence and extent of intimal flap, arterial dissection, plaque ulceration, presence and volume of thrombus (5). IVUS does not only provide diagnostic information per-procedure, but it also may guide the choice of the appropriate PTA method, assist in the accurate deployment of an endovascular device, and check the efficiency of the procedure. In this article, we will discuss the current role and future perspectives of IVUS in the endovascular management of PAD from a practical point of view.

## Technical Aspects

**Figure 1:**
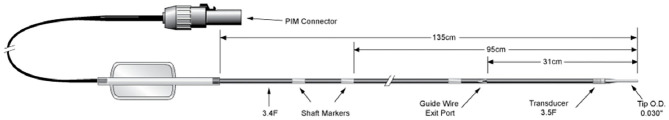
Commonly used peripheral vascular intravascular ultrasound catheter. It comes on a 0.014- or 0.035-inch guidewire with monorail system.

By coupling IVUS catheters with real-time computerized processing devices, this technology has been transformed over the last decade into a user-friendly tool that may give three-dimensional information in order to facilitate endovascular arterial procedures (1, 5). IVUS during vascular interventions offers valuable information about plaque morphology, vessel diameter and lesion severity, lumen area, detection of calcium severity and thrombus, detection of dissections, stent apposition and expansion. IVUS catheters are currently provided by two companies, Philips and Boston Scientific. The catheter sizes range from 2- to 4-French (Fr) and can be easily guided through a 5- or 6-Fr sheath. Larger IVUS catheters are also used for larger peripheral vessel applications and require larger sheaths. The larger IVUS catheters come over 0.035-inch guidewires, and the smaller ones that are more often used for infrainguinal procedures require 0.018-inch or 0.014-inch guidewires, similar to the small-profile balloons or stents used most often in this anatomical location (Figure 1). The length of IVUS catheters ranges from 90 to 150 cm, allowing imaging of infra-popliteal arteries via contralateral approach (5, 6).

IVUS uses a piezoelectric transducer generating sound waves after electrical stimulation at the tip of the catheter. Propagation of the waves into different tissues produces a reflection image based on the acoustic properties of each tissue. First images provided by IVUS technology were in gray-scale and plaque morphology was visually classified based on the echogenicity of the plaque compared to its surrounding adventitia. Thus, 20–40 MHz transducers were routinely used (1, 5). The classification of plaques was then as follows: (1) soft, (2) fibrous, (3) calcified, and (4) mixed plaques. Later, virtual histology-IVUS (VH-IVUS) came up to the market, providing better evaluation and characterization of the histological composition of the arterial plaque thanks to the analysis of an additional low radiofrequency (RF) content. In addition to the gray-scale, this RF signal is processed with an autoregressive model before being matched to color coded histological databases in order to classify plaques based on their morphological composition. Using VH-IVUS plaques may be classified as follows: (1) fibrous tissue, (2) fibro fatty plaque, (3) necrotic core and (4) calcification (1, 6). New RF-based IVUS modalities have evolved including integrated backscattered IVUS (IB-IVUS). They use more sophisticated software algorithms allowing better tissue characterization.

## Current Role of IVUS in Peripheral Arterial Disease

Main data regarding the utility of IVUS in the guidance of lower limb revascularization procedures in patients with PAD and its potential contribution in prolonging the durability of this therapeutic approach are coming mainly from retrospective studies (7). In a systematic review conducted in 2017, Makris et al. identified 13 clinical studies, which evaluated IVUS as an adjunct tool to DSA during endovascular interventions in patients with PAD in iliac, femoral or popliteal arteries (7). The majority were retrospective cohorts with a total number of 2,258 patients having had IVUS as part of their treatment. Out of 13 studies, 7 studies investigated the role of IVUS for PTA and stenting of PAD, 5 studies used IVUS to guide true-lumen reentry and one study tested IVUS during atherectomy. Some more recent studies also investigated the role of IVUS in the management of patients with PAD. Tables 1, 2 summarize characteristics and outcomes of the main studies evaluating IVUS in the endovascular management of PAD.

**Table 1:** Summary of characteristics the main studies assessing IVUS in the endovascular management of PAD.

**References**	**Study design**	**No. of patients**	**Arterial level**	**Procedure type**	Mean follow-up^*^
Buckley et al. (8)	Retrospective	52	Iliac	PTA/primary stenting	62.1
Kawasaki et al. (9)	Prospective	36	Iliac/femoral	Endovascular therapy	9.9
Araki et al. (10)	Retrospective	82	Iliac	Re-canalization/stenting	27.6
Iida et al. (11)	Retrospective	468	Femoral/popliteal	Nitinol stenting	22.8
Baker et al. (12)	Retrospective	40	Iliac/superficial femoral	Re- vascularization	4.3
Kumakura et al. (13)	Prospective	455	Iliac	Stent implantation	63
Panaich et al. (14)	Retrospective	92 714	Peripheral	Peripheral intervention	N/A
Yin et al. (15)	Prospective	47	Peripheral	Atherectomy	N/A
Krishnan et al. (16)	Retrospective	114	Femoro-popliteal in-stent	Directional atherectomy + PTA	12
Shammas et al. (17)	Prospective	15	Femoro-popliteal	Atherectomy	Procedure day
Fujihara et al. (18)	Retrospective	130	Superficial femoral	PTA/stenting	Procedure day
Miki et al. (19)	Retrospective	274	Femoro-popliteal	Stenting	24

*PAD, peripheral arterial disease; IVUS, intravascular ultrasound; No., number; PTA, percutaneous transluminal angioplasty; N/A, not described*.

* *In months*.

**Table 2:** Summary of outcomes of the main studies assessing IVUS in the endovascular management of PAD.

**References**	**Technical success**	**Patency rate**	**Clinical success**	**Complications rate**	**Reintervention rate**
Buckley et al. (8)	N/A	100%	N/A	7%	0% with IVUS
Kawasaki et al. (9)	100%	N/A	N/A	0%	5.6%, no amputation
Araki et al. (10)	N/A	96.5%	N/A	2.4%	No amputation
Iida et al. (11)	N/A	N/A	N/A	N/A	Significantly lower in IVUS group
Baker et al. (12)	90%	62%	N/A	0%	5%
Kumakura et al. (13)	97.2%	89%	N/A	4%	Significantly lower in IVUS group
Panaich et al. (14)	N/A	N/A	N/A	11.8%	IVUS predictive of lower amputation rate
Yin et al. (15)	100%	N/A	N/A	N/A	N/A
Krishnan et al. (16)	100%	82.1%	N/A	0%	Significantly lower in IVUS group
Shammas et al. (17)	100%	100%	N/A	N/A	Dissections better appreciated with IVUS
Fujihara et al. (18)	100%	100%	N/A	N/A	IVUS predictive of lumen gain
Miki et al. (19)	100%	82.5%	N/A	15%	14.6%

*PAD, peripheral arterial disease; IVUS, intravascular ultrasound; N/A, not described*.

### IVUS and PTA/Stenting

Seven studies reported results regarding the role of IVUS for PTA and stenting of PAD (8–14). Four of them were retrospective studies, investigating a total number of 2,258 patients, and comparing PTA and stenting in such a setting with or without the use of IVUS. In total, 1,589 patients were in the IVUS group (8, 11, 12, 14). Long-term patency rates ranged from 62 to 100% in the IVUS group vs. 69 to 83.4% in the non-IVUS group. Follow-up ranged from 4 to 62 months (8, 11, 12, 14). Three of these studies reported free from re-intervention and event free survival data (8, 11, 14). Iida et al. investigated the efficacy of IVUS in femoro-popliteal stenting for PAD with TASC II class A to C lesions (11). The author found a statistically significant difference in favor of the IVUS group in a total of 468 patients. Indeed, IVUS use was associated with a significantly higher 5-year primary patency (65 ± 6% vs. 35 ± 6%, *P* < 0.001) rate, better freedom from any adverse limb event rate (*P* < 0.001) and better event-free survival rate (*P* < 0.001).

Panaich et al. analyzed data from the Healthcare Cost and Utilization Project Nationwide Inpatient Sample on peripheral endovascular procedures performed between 2006 and 2011 (*n* = 92,714; 55% men; mean age, 60 years) (14). Overall, IVUS was used in 1.4% of cases analyzed. IVUS use during lower limb arterial interventions was predictive of lower post-procedural complication and amputation (OR = 0.59; 95%CI, 0.45–0.77; *P* < 0.001) rates with a non-significant increase in hospitalization costs. The overall rate of amputation was 9.7%, with a lower rate in the IVUS group (5.3%) compared to the group without IVUS (9.8%, *P* < 0.001). Baker et al. was the only to report three events of post-procedural complications in the IVUS group (12).

One explanation regarding these results may be the possible superiority and higher accuracy of IVUS compared to DSA alone. Indeed, the evaluation of vessel plaque morphology and vessel size is a major component of peripheral endovascular procedures. Historically, DSA has been considered the gold standard for assessment of vessel size and endovascular treatment. However, DSA has some limitations such as providing a two-dimensional image of a three-dimensional luminal structure (2). It mainly focuses on the lumen and confounding artifacts that derive from the arterial wall motion can be created (4, 20, 21). On the other hand, due to the direct view of the arterial wall, IVUS permits detailed information not only about the lumen, but also about plaque morphology, composition and vessel structure. Furthermore, while DSA underestimates, IVUS evaluates accurately the degree of stenosis and may contribute to the detection of the cause of technical failure (5–7). IVUS does not only provide diagnostic information per-procedure, but it also may guide the choice of the appropriate PTA method, assist in the accurate deployment of an endovascular device, and check the efficiency of the procedure more accurately than DSA.

Pliagas et al. recently conducted a retrospective study on a population of 43 patients who underwent an endovascular treatment using DSA and IVUS imaging modality (22). In total, measurements estimated from DSA images were significantly smaller than those obtained with IVUS imaging analysis. The author concluded that IVUS appears to give a higher degree of accuracy in measuring vessel lumen size. As measurements obtained from DSA under-estimated vessel diameter, the use of IVUS may help in determining the treatment algorithms and lead to improve the endovascular outcomes. IVUS also allows mandatory measurements regarding stent expansion. As a result, it is a powerful tool for the recognition of under deployment, which is the main cause of restenosis (7, 22).

In the three other studies of the meta-analysis, all lower limb revascularization interventions were guided by IVUS (9, 10, 13). Overall, 573 patients were included and follow-up ranged from 10 to 63 months. According to data from the first 5 years after PTA or stenting, long-term patency ranged from 89 to 96.5% and overall survival was from 82 to 100%. Periprocedural complications were reported only by Kumakura et al. in 18 (4%) of the 455 patients (13). Amputation rate was nil in the 3 studies. In addition, Kumakura et al. reported a 71% freedom from MACLE (Major Adverse Cardiovascular and Limb Events) during a 5-year post-procedural period. The favorable long-term patency results from these 3 studies that evaluated only IVUS-guided PTA or stenting also support the efficacy, durability and superiority of this technique, despite the absence of comparative groups. Specifically, Kumakura et al. demonstrated a 5-, 10-, and 15-year patency of 89, 83, and 75%, respectively, with no statistically significant differences among TASC-II categories (13).

### IVUS and Atherectomy

One prospective study assessed the use of IVUS in the guidance of atherectomy in a population of 30 patients and showed an encouraging 100% technical success rate (23). At follow-up, long-term patency was 90% and clinical success was 100%. There were no periprocedural complications or major amputations. However, a revascularization rate of 10% was reported.

More recently, Krishnan et al. retrospectively compared 1-year outcomes for patients with femoro-popliteal in-stent restenosis using directional atherectomy guided by IVUS vs. directional atherectomy guided by DSA (16). Directional atherectomy guided by IVUS reduced clinically driven target lesion revascularization (CD-TLR) for patients with femoro-popliteal in-stent restenosis. Indeed, IVUS in conjunction with directional atherectomy may improve CD-TLR rates for femoro-popliteal in-stent restenosis patients by allowing the operator the ability to more accurately visualize the lesion than with DSA, and thereby minimize residual stenosis post-directional atherectomy treatment through aggressive debulking.

**Figure 2:**
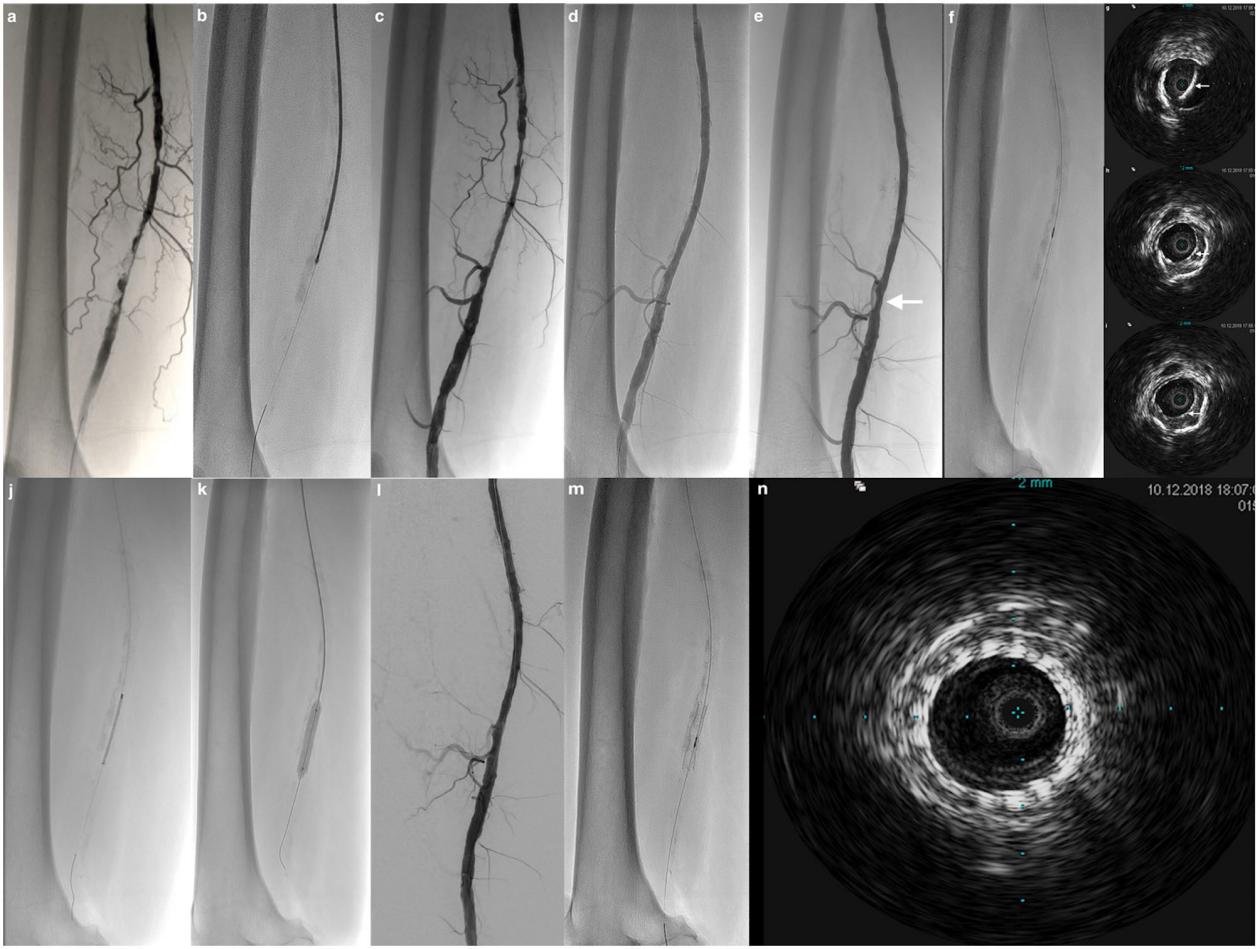
Limping patient of 67-year old. **(a)** Antegrade angiography by common femoral approach shows pre-occlusive calcified stenotic lesion of the mid and distal part of the right superficial femoral artery. **(b)** Atherectomy with the Phoenix device. **(c)** Control after atherectomy shows lumen gain. **(d)** Control after conventional PTA demonstrates no residual stenosis. **(e)** Result after application of drug-coated balloon shows no significant focal residual stenosis (*arrow*). **(f)** Checking with a 0.014-inch IVUS catheter. **(g–i)** IVUS demonstrates very well a focal intimal dissection post-angioplasty at the level of the focal residual stenosis, not visible at angiogram (*arrows*). **(j–l)** Result after spot stenting. **(m,n)** Final IVUS control shows normal arterial lumen with stent patency.

**Figure 3:**
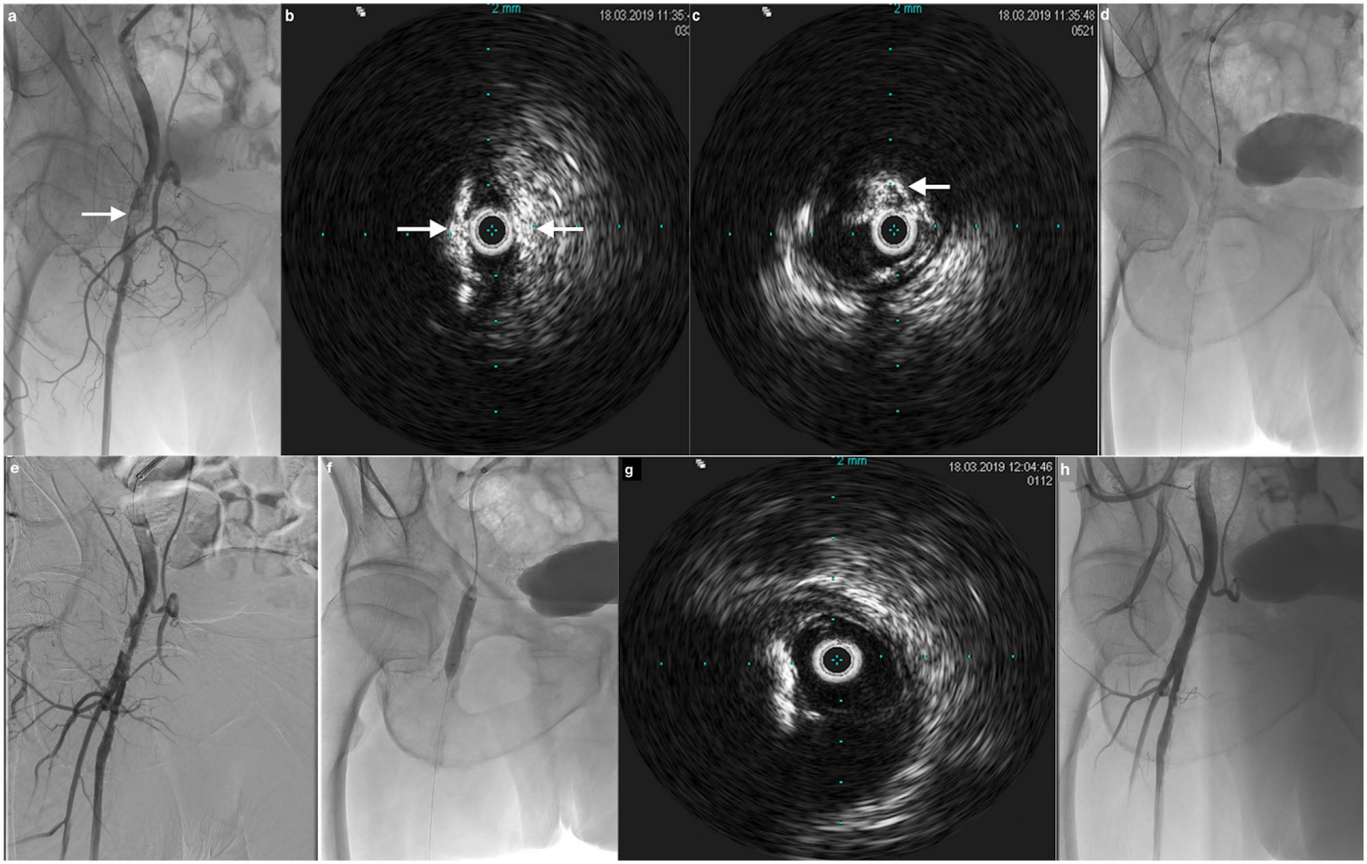
72-year old patient with right claudication. **(a)** Angiogram by crossover shows important calcified stenotic lesions of the right common femoral artery (*arrow*). **(b,c)** IVUS confirms the large circonferential hyperechogenic calcifications (*arrows*). **(d)** Use of JetStream atherectomy device for debulking. **(e)** Result after debulking shows lumen gain. **(f)** Conventional PTA and drug-coated balloon angioplasty. **(g)** IVUS control confirms the excellent debulking result with removal of calcifications and lumen gain. No additional stenting was needed. **(h)** Final result at angiogram demonstrates normal lumen size with no residual stenosis.

Interestingly, results from the CliRpath Excimer Laser System to Enlarge Lumen Openings (CELLO) registry including patients treated with specific excimer laser systems for the vascular management of PAD affecting the superficial femoral and proximal popliteal arteries were recently reported (24). The goal of this study was to evaluate, via IVUS, the dissections in the arterial wall after treatment with the laser devices. Treatments using the specific Excimer laser catheters resulted in a significant increase in lumen area of 5.5 ± 3.2 mm^2^ (95%CI, 4.3–6.8, *P* < 0.0001) and reduction in the volume of plaque plus media of −10.6 ± 36.0 mm^3^ (95%CI, −25.8 to 4.6, *P* = 0.1619) whilst giving rise to intramural hematoma after Turbo-Booster laser therapy in 55% of frames evaluated and 24% medial dissections with <1% of adventitial disruption. The Excimer laser-based Turbo-Booster management of PAD resulted in significant plaque removal and increased lumen size with almost no adventitial layer injury (24). Figures 2, 3 illustrate the usefulness of IVUS after atherectomy.

### IVUS and True-Lumen Re-entry

Five retrospective studies, that evaluated IVUS for true-lumen reentry during subintimal PTA, showed very promising technical success rates ranging from 97 to 100%, whatever the catheter used (9, 25–29). Follow-up demonstrated a clinical success rate of 100% in 3 of the studies (25, 27, 29). The goal of one of these 5 studies was to perform comparisons between true lumen re-entry with and without the use of IVUS (26). Technical success was higher in the IVUS group (97 vs. 81%). The real time imaging of IVUS enables subintimal tract creation and directed needle deployment. IVUS scanner adds information not only about the intima and the lumen, but also confirms vessel patency at the point of the needle due to its color flow capabilities. The accuracy and controlled reentry offered by IVUS catheters reduce the risk of dissection-related complications, such as perforations caused by guidewire or catheter malpositions, even for thick calcified plaques (6, 7, 25). Another advantage of IVUS reentry catheters is that they shorten intervention time between 3 and 10 min. This limits the radiation exposure for the patient and operator (27, 29). In addition, the use of these devices decreases the risk of periprocedural complications that are related to prolonged operation times. As a result, in the 5 studies that investigated the use of IVUS reentry devices, no significant periprocedural or post-procedural complications were reported.

### IVUS and Contrast Exposure

In 2 studies, it was proposed that with IVUS guidance contrast injection could be reduced or completely avoided (9, 26). Kawasaki et al. performed comparisons between IVUS and non IVUS guided true-lumen re-entry and demonstrated a significantly lower contrast exposure in favor of the IVUS group (104 vs. 201 mL, *P* < 0.001) (26). Another benefit of IVUS is that injection of contrast medium can be reduced or even completely avoided, as it has been reported in many studies (9, 26, 30, 31). Contrast induced acute kidney injury may be associated with higher morbidity and mortality rates as well as prolonged hospitalization. Therefore, this contrast-free alternative seems to be very promising for high risk patients suffering from diabetes, chronic renal insufficiency or contrast allergies, but also for all other patients due to the contrast overload reduction.

### IVUS and Cost-Effectiveness

Two studies suggested that IVUS leads to additional cost to a revascularization procedure, with an increase that ranges from $1,080 to $1,333 (8, 14). According to Panaich et al. the IVUS derived increase of procedural costs was non-significant ($1,333, 95%CI,–$167 to + $2,833, *P* = 0.082) (14).

Buckley et al. reported that the medical costs were higher with IVUS guidance vs. DSA alone during PAD interventions (8). According to Schiele et al. acute procedural costs can be 18% higher with IVUS compared to non IVUS use (32). However, the cumulative costs are only slightly higher ranging from 1 to 7.8% due to the lower number of re-interventions and the decreased length of hospital stay in the IVUS guided endovascular procedures, meaning that the use of IVUS can be cost effective when properly used.

## Future Perspectives

Although retrospective studies report a high rate of patency and freedom from revascularization with the utilization of IVUS imaging as an adjunctive tool to standard DSA, data from randomized controlled trials are still lacking. Evaluation and improvement of stent placement in PAD and the identification of post-procedural dissections that are missed by DSA alone can be obtained with the use of IVUS (7, 33). Despite a significant improvement in immediate technical outcomes and short-term results, wide adoption of this technology prior to peripheral interventions requires universal algorithm to optimize long-term outcomes. In addition, IVUS enables to diminish the amount of contrast medium used and its associated complications in peripheral endovascular interventions (26, 31). Last, despite cost-effective analyses in favor of the use of IVUS by decreasing the rate of complications in lower limb interventions, further studies are needed to definitely demonstrate the cost-effectiveness of the systematic and routine use of IVUS during endovascular interventions. Indeed, the use of disposable catheter-delivered transducers, the expensive equipment and an experienced technician who may be necessary for the operation of IVUS system are the main factors responsible for the increased procedural costs of this technique (6, 8). However, there is no doubt that IVUS is likely to be more often used in the near future for modern endovascular therapy in PAD.

## Summary and Conclusions

IVUS has improved fast from a purely diagnostic imaging modality to a very useful adjunctive tool to DSA in the evaluation of the vasculature. It may provide a more precise visualization to what happens into the blood vessel and play an increasing role in peripheral arterial occlusive procedures. Miniaturization of the components has allowed the system and catheter to be as small-profile as new technologies, and has made IVUS a mandatory adjunct tool for the best outcomes. As endovascular procedures become increasingly complex, technical, and clinical successes will be related to the degree of accuracy of the guidance device used during the procedure. IVUS is relatively easy to use and can be widely available despite cost but requires some expertise in image interpretation which might limit its systematic use. However, IVUS is an important element of current and future vascular interventions and should be part of training programs and the routine practice of all vascular specialists.

## Author Contributions

RL, NF, CG, LF, FL, MM, and OC the conception and design of the study, or acquisition of data, or analysis and interpretation of data, drafting the article or revising it critically for important intellectual content, and final approval of the version to be submitted. Each author has participated sufficiently in this article to take public responsibility for its content.

## Conflict of Interest

The authors declare that the research was conducted in the absence of any commercial or financial relationships that could be construed as a potential conflict of interest.
